# An unusual case of lower trunk brachial plexus zoster reactivation

**DOI:** 10.1080/23320885.2023.2242497

**Published:** 2023-08-02

**Authors:** Samantha J. Burch, Elizabeth Shepard, Angelo B. Lipira

**Affiliations:** Division of Plastic and Reconstructive Surgery, Oregon Health and Science University, Portland, OR, USA

**Keywords:** Herpes zoster, lower trunk, zoster reactivation

## Abstract

We discuss the case of a 42-year-old woman who presented with severe left cubital tunnel neuropathic pain and subsequently developed a vesicular rash spanning the C8-T1 dermatomal distribution. These symptoms resolved after initiation of acyclovir, highlighting VZV brachial plexopathy as a potentially treatable etiology of acute onset severe neuropathic pain.

## Introduction

Herpes zoster, or shingles, is a common condition caused by reactivation of the varicella-zoster virus (VZV), a double-stranded DNA alphaherpesvirus [1]. Shingles is characterized by a two to three day prodrome of neuropathic pain prior to the eruption of vesicular skin lesions, although cutaneous findings are not required for diagnosis [2]. These skin lesions classically present in a dermatomal pattern, as VZV remains latent in the dorsal root ganglia after primary infection. While VZV primarily affects sensory nerves, motor weakness may be present in 1–5% of patients with herpes zoster [3]. Segmental Zoster Paresis involves motor weakness in the same dermatomal distribution as the vesicular rash, most commonly affecting the face and upper extremity [4]. Motor symptoms in the upper extremities following herpes zoster can also be classified as a mononeuropathy, radiculopathy, or brachial plexopathy, which are confirmed and monitored by electrophysiologic testing or MRI [3]. In most cases, neuropathic pain and motor symptoms resolve with time, however a subset of patients can experience postherpetic neuralgia (PHN) or persistent motor deficits, for reasons that remain poorly understood [5].

We present the case of a 42-year-old who presented with five days of severe left cubital tunnel pain and ulnar sided hand paresthesias, ipsilateral acute shoulder pain, and a vesicular rash in the C8-T1 distribution which resolved with antiviral treatment– consistent with a clinical diagnosis of lower trunk herpes zoster. An unusual aspect of this case was that it presented with pain without objective neurological deficits.

## Case description

A 42-year-old otherwise healthy woman presented to hand surgery clinic with five days of acute onset severe pain over her left cubital tunnel, three days of ipsilateral shoulder pain, and one day of a vesicular rash along her ulnar palm and dorsal ulnar forearm. She was without recent injury, immunization, or illness. Her history was notable for self-limited varicella zoster infection in childhood. Prior to this episode she endorsed intermittent episodes of nighttime waking with paresthesia of her ring and small finger but did not carry a diagnosis of cubital tunnel syndrome.

Her physical exam was notable for the presence of a clustered vesicular rash over her hypothenar eminence and dorsal ulnar forearm which she said only appeared that day. She had trace edema, and severe tenderness to palpation over the ulnar nerve at the left cubital tunnel without overlying rash. She had no objective loss of strength of the upper extremity and was without intrinsic weakness or clawing. Notably, her cubital tunnel compression (elbow flexion test) and Tinel’s tests were positive, though she displayed normal two-point discrimination across all fingers.

**Figure 1.**
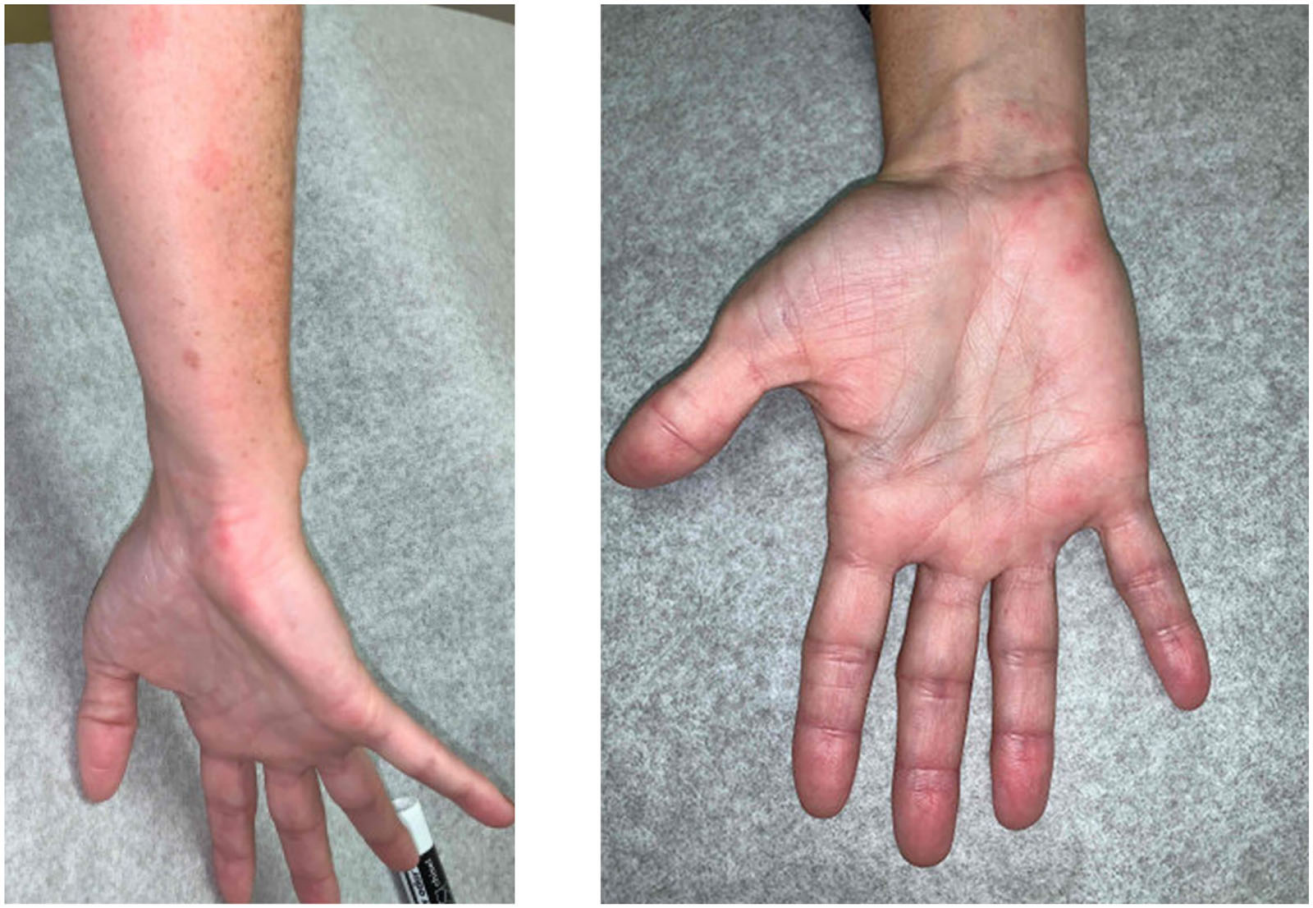
A 42-year-old woman presented with neuropathic pain and Subsequent eruption of vesicular rash in the C8-T1 dermatomal distribution, as pictured.

Initial differential diagnosis included Varicella zoster reactivation of the brachial plexus lower trunk versus Parsonage Turner syndrome (PTS). She was treated with one week of Acyclovir. Given lack of objective motor or sensory deficits the decision to proceed with EMG, MRI or urgent cubital tunnel decompression was deferred. Objective findings were notable for persistent cubital tunnel swelling and a positive cubital tunnel compression test. Within two weeks of initiating acyclovir, she experienced significant improvement in her symptoms. As seen in Figure 1, self-documented photographs demonstrated that the rash was most prominent at one week, fully spanning the C8-T1 dermatomal distribution.

## Discussion

This case report details an unusual presentation of herpes zoster involving the lower trunk of the brachial plexus, characterized by acute ipsilateral shoulder pain followed by neuropathic pain in and then eruption of a vesicular rash along the C8-T1 dermatomes.

Cases of lower trunk brachial plexopathy associated with zoster reactivation have rarely been described in the literature. Those reported have generally been associated with moderate to severe sensory or motor impairment [6,7]. Segmental zoster paresis is a rare complication of herpes zoster characterized by motor weakness in the corresponding region of the vesicular rash [4]. Kesserwani described a case of C8 zoster reactivation with weakness of the left hand involving the FPL, APB, and first dorsal interosseus muscles [6]. Yamada described a similar case of weakness of the C8-T1 myotomes and sensory impairment spanning C5-T1 following herpes zoster lesions in the upper, middle, and lower trunks of the brachial plexus [7]. Kawajiri et al. described three such patients with brachial plexopathies related to herpes zoster, two of which experienced weakness of the shoulder with a corresponding rash in C5 and C6 dermatomes [8]. Our patient experienced pain without motor weakness in the C8-T1 and ipsilateral posterior scapular pain.

A case report published by Cukic is most similar to our patient, describing a woman with acute pain of the right scapula, elbow and the ulnar aspect of the forearm who later developed a hemorrhagic vesicular rash in the distribution of the ulnar nerve [5]. Their patient continued to experience intermittent symptoms of pain after vesicle resolution unlike our patient who had resolution of her pain soon after acyclovir treatment. Cervical radiculopathy has been described in association with positive history of VZV reactivation and could be a long-term complication. Gumina et al. studied 110 patients with shoulder pain as a result of cervical radiculopathy, finding that 34.5% had a history of VZV reactivation [9]. Whether these patients developed the shoulder pain before the lesions is not clear.

Initially, due to the ipsilateral shoulder discomfort, a diagnosis of Parsonage Tuner Syndrome (PTS) was considered. PTS, also referred to as brachial plexus neuritis or neuralgic amyotrophy, typically presents as severe pain across the shoulder preceding weakness of the muscles of the shoulder, arm, wrist, or hand [10]. The mechanism remains incompletely understood, but many studies attribute PTS to an immune-mediated response to triggers such as exertion, pregnancy, or surgery [11]. Recent studies have identified hourglass-like fascicular contractions on imaging studies which were confirmed intra-operatively as well as inflammation with perivascular lymphoid cells on perineurial biopsies. One study by ArAnyi et al. found 30% of patients with PTS had an event of mechanical stress within 3 weeks of symptom onset [12]. While our patient did demonstrate some signs and symptoms of PTS, it became evident at the two-week interval that her primary process was VZV reactivation. This case highlights potential overlap of presentation of PTS symptomatology and viral mediated neuritis.

Nucleoside analogues such as acyclovir, valacyclovir, or famciclovir have replaced vidarabine and interferon alpha as the standard treatment for Herpes Zoster, most effective when initiated within 72 h of rash onset [13,14]. Acyclovir 800 mg five times daily for seven days continues to be the mainstay of treatment for many providers [13]. Acyclovir is phosphorylated by the VZV thymidine kinase and metabolized to a competitive inhibitor of viral DNA polymerase [14]. The bioavailability of this drug is 15–30%, increased to 54% with the addition of oral Valacyclovir, the prodrug of Acyclovir. Some clinicians favor prodrugs such as Valacyclovir and Famciclovir to Acyclovir as these medications have improved bioavailability and require decreased dosage frequencies [15,16]. While these three drugs are comparable in their ability to achieve resolution of symptoms, an important patient consideration is that Valacyclovir and Famciclovir are more expensive regimens [14]. Systemic corticosteroids can be used in addition to antivirals in special circumstances of zoster infection including ocular or facial nerve involvement, though their overall efficacy in altering the course of brachial plexus neuropathy or preventing post herpetic neuralgia remains unclear [17]. There may be a role for other medications such as topical lidocaine, pregabalin, tricyclic antidepressants, SNRIs or gabapentinoids, although the efficacy of these medications is variable in the literature [18]. Lastly, incidence of PHN in patients with Herpes Zoster ranges in the literature between 9 and 73% but a statistically significant decrease in incidence has been shown when antiviral treatment is initiated within 72 h of rash onset [19].

In conclusion, we describe a patient with acute pain of the shoulder who subsequently developed a vesicular rash along the C8-T1 dermatomes. While previous studies have described brachial plexus involvement by herpes zoster, generally these cases are associated with weakness or sensory impairment. Interestingly, our patient had pain as the primary manifestation, likely in part due to nerve edema and secondary compression of the ulnar nerve within the cubital tunnel. It is important to acknowledge that while VZV is a clinical diagnosis, our case report has several limitations including lack of EMG, nerve conduction studies, MRI imaging or VZV serology. These diagnostic studies were deferred as they were not clinically indicated or necessary for appropriate treatment for this patient but were a part of her plan if she didn’t promptly improve. Nevertheless, we believe our case report adds value to the literature given the paucity of information regarding brachial plexus VZV neuropathy. Our patient’s symptoms completely resolved within weeks after initiation of acyclovir. Fortunately, this patient happened too present to our clinic on the day the rash first appeared, allowing prompt initiation of treatment. This case highlights a potentially treatable etiology that should be on the differential of acute onset severe neuropathic pain.
